# Increasing immunoglobulin G adsorption in dextran‐grafted protein A gels

**DOI:** 10.1002/elsc.202000097

**Published:** 2021-03-20

**Authors:** Liming Huan, Qing‐Hong Shi

**Affiliations:** ^1^ Department of Biochemical Engineering School of Chemical Engineering and Technology Tianjin University Tianjin P. R. China; ^2^ Key Laboratory of Systems Bioengineering and Frontiers Science Center for Synthetic Biology (Ministry of Education) Tianjin University Tianjin P. R. China

**Keywords:** carboxymethyl dextran, grafting density, IgG adsorption, ligand density, protein A chromatography

## Abstract

The formation of a stable spatial arrangement of protein A ligands is a great challenge for the development of high‐capacity polymer‐grafted protein A adsorbents due to the complexity in interplay between coupled ligands and polymer chain. In this work, carboxymethyl dextrans (CMDs) with different molecular weight were introduced to provide stable spatial ligand arrangement in CMD‐grafted protein A gels to improve IgG adsorption. The result showed that coupling of protein A ligand in CMD‐grafted layer had no marked influence on pore size and dextran layers coupled with the ligands were stable in experimental range of salt concentrations. The result of IgG adsorption revealed that carboxymethyl dextran T10, a short CMD, was more suitable as a scaffold for the synthesis of high‐capacity protein A gels. Moreover, the maximal adsorption capacity for IgG was obtained to be 96.4 mg/g gel at ionic capacities of 300–350 mmol/L and a ligand density of 15.2 mg/g gel. Dynamic binding capacity for IgG exhibited a higher capacity utilization in CMD‐grafted protein A gels than non‐grafted protein A gel. The research presented a tactics to establish a stable dextran layer coupled with protein A ligands and demonstrated its importance to improve binding capacity for IgG.

**Abbreviations**
4FF: Sepharose 4FF
6FF: Sepharose 6FF
CMD: carboxymethyl dextran
CMD10: carboxymethyl dextran T10
CMD40: carboxymethyl dextran T40
CMSep: CM Sepharose
DBC: dynamic binding capacity
EDC: *N*‐(3‐dimethylaminopropyl)‐*N'*‐ethylcarbodiimide hydrochloride
IC: ionic capacity
iSEC: inverse size exclusion chromatography
*K*_*d*_: dissociation constant
PDEA: 2‐(2‐pyridyldithio) ethylamine hydro‐chloride
*q*_*m*_: saturated adsorption capacity
Tris: tris(hydroxymethyl)aminomethane
Z_1_: monomeric Z domain
Z_2_: dimeric Z domain
Z_4_: tetrameric Z domain

## 1 INTRODUCTION

Since the first monoclonal antibody (mAb) was approved in 1986 [1, 2], several typical platform technologies have been established for commercial mAb production due to the similarities in structure, physico‐chemical properties and regulatory requirement of mAb molecules [3, 4, 5]. In downstream processing, protein A chromatography followed by two polishing chromatographic techniques have been employed for purifying mAb [5, 6, 7]. In this protocol, protein A chromatography serves as core platform techniques for antibody purification and has become the golden standard in mAb production pipeline [8]. This technique was firstly commercialized in 1978 [9]. Early version of protein A adsorbent always suffered from serious problems in low binding capacity and weak stability. The growth in capacity and productivity of protein A chromatography lags seriously behind upstream processing. In contrast to an annual growth of 14% in mAb bioreactor titer, the productivity of protein A adsorbents had an annual growth of 4.3% as reported by Bolton and Mehta [10]. Capacity bottleneck is a great challenge for industrial application of protein A chromatography.

In 1987, Nilsson *et al*. synthesized an engineered protein A ligand mutated from B domain (named as Z domain) by the G29A mutation [11]. Compared with native B domain, the engineered Z domain not only maintained the same antibody affinity as B domain, and but was more stable in alkali solution. Its stability further increased with an increase in polymerization degree of the domain. So far, engineered Z domain and its derivatives is the most frequently used ligands in commercial protein A chromatography. In protein A chromatography, binding capacity for antibody was influenced by many factors including ligand types [12, 13, 14] and density[15, 16, 17, 18], immobilization techniques [13, 17, 18], matrix properties [15, 19, 20], operation conditions [21] and molecular orientation of adsorbed antibody [22]. Adsorption capacity for antibody is largely dependent on ligand density of protein A adsorbents [13, 16, 17, 23]. The dependence of ligand density has ever been reported in ion exchange chromatography and affinity chromatography [24, 25, 26, 27]. Černigoj *et al*. found that dynamic binding capacity for antibody grew with an increase in density of protein A ligands [23]. Ghose *et al*. also revealed that adsorption capacity for antibodies reduced markedly as ligand density decreased by over 50% on Prosep A Ultra High Cap, a commercial protein A adsorbent from Millipore [16]. On the other hand, there was no marked change in adsorption capacity for antibody as ligand density decreased by 25%. It indicated that adsorption of protein A adsorbents were limited by surface area of the matrices. It is well known that specific binding induces the formation of a monolayer of adsorbed antibody on solid surface of protein A adsorbents. Once the density of protein A ligand reached to a critical level, a further increase in ligand density did not work and the ligand utilization decreased greatly instead. Therefore, available surface area of matrices for antibody binding is another dominant factor for adsorption capacity [22, 28], and decreased greatly with an increase of protein size in a given matrix [29].

**PRACTICAL APPLICATION**

This work presented a tactics to establish a stable dextran layer coupled with protein A ligand in pores of Sepharose gels to improve adsorption capacity for IgG in protein A chromatography. It was achieved by the grafting of charged carboxymethyl dextran (CMD) onto Sepharose 4FF and Sepharose 6FF gels, and universal for chromatographic matrices of various origins. The result showed that coupling of protein A ligand in CMD‐grafted layer had no marked influence on pore size and dextran layers coupled with the ligands were stable in experimental range of salt concentrations. Moreover, a shorter CMD T10 was more suitable as a scaffold than CMD T40 for the synthesis of high‐capacity protein A gels. By optimizing the synthesis of CMD‐grafted protein A gels, adsorption capacity for IgG reached to 96.4 mg/g gel at a ligand density of 15.2 mg/g gel.

Binding capacity of protein A adsorbents can also be improved by the application of linear polymerized protein A ligands [14, 17, 18]. Ghose *et al*. found that each protein A ligand containing five domains for antibody binding just could bind 2.4–3.1 antibodies or Fc‐fusion proteins in solution [16]. During isothermal titration calorimetric measurement, Yang *et al*. further validated that binding stoichiometries of antibody was 2.5 for tetrameric Z domain and 3.0 for hexameric Z domain whilst each monomeric and dimeric Z domains just bind one antibody molecule [18]. The influence of the domain polymerization was likely reflected on protein A adsorbents [13, 16, 17, 18]. As reported by von Roman *et al*. [17], adsorption capacity for antibody grew with an increase in the polymerization of B domains from protein A at the similar molar densities. Recently, the result in our group further showed that adsorption capacity for antibody hexameric protein A ligand was lower than that of tetrameric protein A ligand [18]. With an increase of polymerization degree of protein A ligand, part of domains in protein A ligand was gradually far away from the matrix surface and it bound to antibody in pore space, rather than on matrix surface. As a result, the utilization of pore space increased and the steric exclusion effect of the matrix was eliminated. Compared with the improvement of adsorption capacity, an increased polymerization degree as well as ligand density always led to a decreased utilization of binding sites in protein A ligand [17, 18, 23].

Evidences of ligand polymerization give a valuable clue to improve binding capacity by making better utilization of pores space in protein A adsorbents. The idea has extensively been applied in polymer‐grafted ion‐exchange adsorbents [24, 30, 31, 32, 33] and also reported in polymer‐grafted mixed mode chromatography [34, 35, 36, 37]. Our previous research showed that polymer‐grafted ion‐exchange adsorbent had much high adsorption capacity for proteins approaching to the upper limit of protein stacking in pore space with a cubic dense lattice by adjusting chain length and grafting density [33]. Gu *et al*. further found that dextran T5‐grafted mixed mode gel had similar adsorbed amount for proteins to non‐grafted mixed mode gel and both of them were much smaller than the adsorbed amount of dextran T20‐grafted mixed mode gel [34]. Zhao *et al*. synthesized a dextran‐grafted protein A adsorbent by coupling ligand onto dextran chain and the adsorbent increased binding capacity by 24% compared with non‐grafted protein A adsorbent [38]. However, polymer grafting and ligand coupling in polymer layer always had a significant influence on pore architecture of the matrices. A shrinkage of pore size is the common feature in polymer‐grafted ion exchange adsorbent [30, 39, 40]. Moreover, our group found previously that coupling of mixed‐mode and octapeptide ligands induced hydrophobic collapse of dextran‐grafted layer in pores of Sepharose FF [35, 41]. As reported by Xue *et al*. [41], dextran‐grafted octapeptide affinity Sepharose FF gel had lower adsorption capacity for antibody than non‐grafted octapeptide affinity gel at the same ligand density and adsorption capacity decreased with an increase of ligand density. In the view of the complexity of the interplay between protein A ligand and neutral dextran chain, dextran‐grafted protein A adsorbents may face the same trouble in the stability of dextran layer and spatial arrangement of protein A ligand. Therefore, it is a great challenge for researchers to provide a stable polymer layer for the coupling of protein A ligands.

To achieve this goal, we attempt to synthesize polymer‐grafted matrices for protein A adsorbent by grafting charged carboxymethyl dextran (CMD) with different molecular on the matrices, Sepharose 4FF and Sepharose 6FF gels. After three protein A ligands, monomeric, dimeric and tetrameric Z domains, were coupled onto CMD‐grafted gels, the influence of pH, ionic strength and ligand density on the performance of CMD‐grafted protein A gels was evaluated in detail. The results were compared further with those of non‐grafted protein A gel using CM Sepharose FF as the matrix. This research proposed a possibility and made an attempt to form a stable polymer layer for coupling of protein A ligand in the development of high‐capacity protein A chromatography.

## 2 MATERIALS AND METHODS

### 2.1 Materials

Sepharose 4FF gel, Sepharose 6FF gel, CM Sepharose gel, Sephadex G25, Tricorn 5/50 and HK 16/20 columns, and pre‐packed HisTrap HP column (5 mL) were the products of GE Healthcare (Uppsala, Sweden) and received from local supplier. IgG (*M*_w_ ∼ 150 kDa, purity >99%), *N*‐hydroxysuccinimide (NHS), blue dextran (*M*_w_ ∼ 2000 kDa), CMD T10 (*M*_w_ ∼ 10 kDa, CMD10) and CMD T40 (*M*_w_ ∼ 40 kDa, CMD40) were purchased from Sigma‐Aldrich (St. Louis, Missouri, USA). Tryptone and yeast extracts were obtained from Oxoid (Hant, UK). Glucose and dextran standards were received from the Chinese Academy of Metrology (Beijing, China), and their basic properties are shown in Table S1. *N*‐(3‐dimethylaminopropyl)‐*N*'‐ethylcarbodiimide hydrochloride (EDC) was obtained from J&K Science Co., Ltd. (Beijing, China). 2‐(2‐pyridyldithio) ethylamine hydrochloride (PDEA) was obtained from Shanghai Bide Pharmaceutical Technology Co., Ltd. (Shanghai, China). Tris(hydroxymethyl)aminomethane (Tris), isopropyl‐*β*‐d‐thiogalactoside (IPTG) and kanamycin sulfate were received from Shengong Bioengineering Co., Ltd. (Shanghai, China). Epichlorohydrin (ECH), sodium hydroxide and all other chemicals are of analytical grade from local sources.

In this work, mono‐, di‐ and tetra‐meric Z domain was prepared as the ligands for protein A adsorbents, and named as Z_1_, Z_2_ and Z_4_, respectively. As shown in Figure S1, an additional CK tag was introduced at C‐terminus of the ligands for the oriented immobilization via thiol group in cysteine. The BL21 *E*. *coli* strains transfected with pET‐30a plasmids encoding the gene for Z_1_, Z_2_ and Z_4_ comprising six histidine at the amino terminus (called respectively as pET‐30a(+)−Z_1_, pET‐30a (+)−Z_2_ and pET‐30a(+)−Z_4_ plasmids) were received from Sangon Biotech Co., Ltd (Shanghai, China).

### 2.2 Expression and purification of protein A ligands

The acquisition of protein A ligands in this work is slightly different from the method described by Yang *et al*. [18]. *E*.coli BL21 strains, respectively, with pET‐30a(+)−Z_1_, pET‐30a(+)−Z_2_ and pET‐30a(+)−Z_4_ plasmids were inoculated in 20 mL LB medium containing 50 mg/L kanamycin sulfate at 37°C and 170 rpm overnight. Then, an aliquot of seed culture was transferred into 1 L Luria Bertani (LB) medium with 30 mg/L kanamycin sulfate, the cell was grown to an OD_600_ of 0.6, at which IPTG with a final concentration of 0.5 mmol/L was added to induce protein expression. After induction for 6 h, the cell was harvested by centrifugation at 4500 rpm and 4°C for 25 min. The collected precipitate was resuspended in binding buffer (50 mmol/L Tris‐HCl, 150 mmol/L NaCl, 20 mmol/L imidazole, pH 7.4). After the cell was lysed by sonication in an ice‐water mixture (2 s pulse with 3 s intervals) for 15 min, the insoluble debris was removed by centrifugation at 5000 rpm for 35 min. The supernatant was then applied directly onto a HisTrap HP column precharged with nickel ion. The target protein was eluted by 150 mmol/L NaCl and 300 mmol/L imidazole in 50 mmol/L Tris‐HCl buffer (pH 7.4). The eluted fraction was desalted through a HK 16/20 column packed with Sephadex G25 and freeze‐dried for subsequent experiments.

### 2.3 Synthesis of CMD‐grafted Sepharose gels and ligand coupling

**FIGURE 1:**
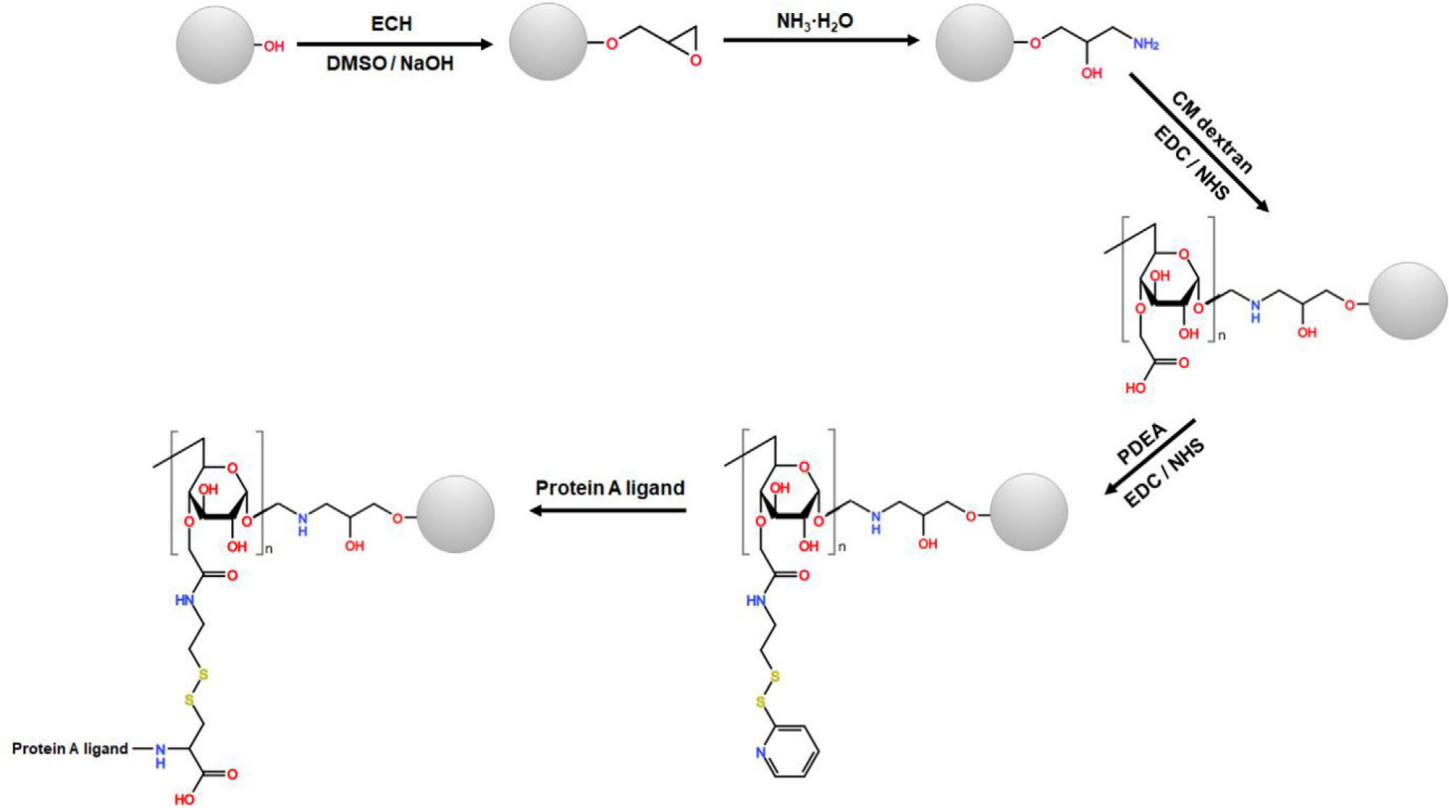
Synthesis of CMD‐grafted Sepharose gel and protein A gels

CMD‐grafted protein A gels were prepared by coupling protein A ligands onto CMD‐grafted Sepharose gels. The CMD‐grafted Sepharose gels were synthesized as described previously with a modification as presented in Figure 1. After Sepharose gels were rinsed by water‐dimethyl sulfoxide (DMSO) mixtures with an increased DMSO gradient of 25, 50, 75% and pure DMSO, the drained gel was mixed with 2 mL ECH, 4 mL DMSO and 4 mL 1 mol/L NaOH, and reacted at 45°C and 170 rpm for 4 h. The product was washed to remove free reactants and mixed with 1.5 volumes of concentrated aqueous ammonia at 45°C and 170 rpm for 4 h. The ammoniated gel was washed with excess water to remove free ammonia, and then mixed with 1 mL CMD solution at 25℃ and 170 rpm for 12 h. After that, EDC and NHS with mass ratio of 4:1 were added and the reaction was continued at 25°C and 170 rpm for 24 h. The CMD‐grafted Sepharose gel was washed with excess water and stored for ligand coupling. In this work, Sepharose 4FF and Sepharose 6FF gels were applied for the synthesis of CMD‐grafted Sepharose gels by grafting of CMD10 and CMD40, respectively.

Protein A gel was prepared by coupling protein A ligands onto CMD‐grafted Sepharose gel as described by Yang *et al*. with a modification [18]. As a reference, non‐grafted protein A gel was prepared on CM Sepharose gel. In brief, 1 g of the drained CMD‐grafted gel was mixed with 0.4 mol/L EDC and 0.1 mol/L NHS (5 mL, v/v = 1:1) and the mixture was reacted at 25℃ and 170 rpm for 0.5 h. After washing with deionized water, 5 mL PDEA solution (160 mmol/L PDEA, 100 mmol/L NaCl, 20 mmol/L PB, pH 8.0) was added and the mixture was reacted at 25℃ and 170 rpm for 1 h. Then, 2 mg/mL ligand in 20 mmol/L PB buffer containing 100 mmol/L NaCl (pH 4.5) was added and the mixture was reacted at 25°C and 150 rpm for 2 h. Finally, the supernatant was collected, and residual ligand concentration was analyzed with an Elite Supersil C4 HPLC column (Dalian, China) at 280 nm as described by Yang *et al*. [18]. The ligand density of protein A gels was calculated based on mass balance. Residual mercaptopyridine on the gel was deactivated by cysteine. The product was washed with excess deionized water to remove residual reagents. In this work, three domains, Z_1_, Z_2_, and Z_4_, were used as protein A ligand.

### 2.4 Characterization of CMD‐grafted protein A gels

In this work, ion exchange capacity (IC) values, volume‐weighted average diameters of the resins (*d*_p_) and densities of hydrated resins (*ρ*_p_) of the gels were measured by the methods described by Yu *et al*. [39]. Each measurement was carried out in triplicate and the average values were represented with its standard deviation. Inverse size exclusion chromatography (iSEC) was introduced to characterize the pore size distributions of CMD‐grafted and non‐grafted gels as described by Li *et al*. with a minor modification [42]. In brief, Tricorn 5/50 column packed with the gels to be measured was connected to Agilent 1100 HPLC Series chromatography in combination with a refractive index detector (RID) (Santa Clara, CA, USA). In the measurement, 20 mmol/L phosphate buffer (pH 7.4) was used as the mobile phase. After the column was equilibrated with mobile phase at 1 mL/min, a series of 2 mg/mL probe solution (20 μL) were injected and the retention volume (*V*_R_) of each probe was recorded. The pore size radius (*r*_pore_, nm) of the gels was calculated based on a single cylindrical pore model according to the method described by Yu *et al*. [39].

### 2.5 Adsorption equilibria and column chromatography

IgG adsorption isotherms on the non‐grafted and CMD‐grafted protein A gels were determined in 20 mmol/L phosphate buffer at different pHs (pH 4.5, 7.4 or 10.0) and NaCl concentrations (0−150 mmol/L) as described by Yang *et al*. [18]. Saturated adsorption capacity (*q*_*m*_, mg/g gel) and dissociation constant (*K*_*d*_, mg/mL) of IgG were obtained by fitting the experimental data to Langmuir model as follows,

(1) $$q=\frac{q_{m}c}{K_{d}+c}.$$

Dynamic binding capacities (DBC) for IgG onto the non‐grafted and CMD‐grafted protein A gel were measured in a Tricorn 5/50 column connected to an ÄKTA Start (GE Healthcare, Uppsala, Sweden). In the experiment, 100 mmol/L NaCl in 20 mmol/L phosphate buffer (pH 10.0) was applied as binding buffer, and the preparation of IgG solution. After the column was equilibrated with binding buffer, 0.5 mg/mL IgG solution was loaded at 0.5 mL/min. After IgG breakthrough, the column was washed with binding buffer to remove free protein and the adsorbed IgG was eluted by 0.01 mol/L glycine‐HCl buffer (pH 3.0). Finally, the column was regenerated by 0.1 mol/L NaOH for next experiments. DBC for IgG was calculated as follows,

(2) $$\mathrm{DBC}=\frac{c_{0}\left(V_{10}-V_{0}\right)}{V_{B}}$$

where *V*_10_ and *V*_B_ are loading volume at 10% breakthrough and the column volume, respectively; *V*_0_ is the dead volume measured with 2% acetone aqueous solution.

## 3 RESULTS AND DISCUSSION

### 3.1 Properties of CMD‐grafted protein A gels

In this work, eleven CMD‐grafted Sepharose gels were synthesized with different ionic capacities (ICs) using two matrices, Sepharose 6FF and Sepharose 4FF, and two types of CM dextran, CMD10 and CMD40. For each CMD‐grafted Sepharose gel, IC values correspond to amount of grafted CMD on the gels. The characteristics of CMD‐grafted Sepharose gels are listed in Table 1. It showed that CMD grafting did not led to a marked change in the size and density of the gels. It was consistent with the results in previous literature [39, 40, 43]. As listed in Table 1, Sepharose 4FF and Sepharose 6FF had average pore radii of 21.26 and 17.57 nm, respectively. CMD grafting led to a shrinkage of pore size of the gels, and CMD40‐6FF‐IC260 had the minimal pore radius of 12.24 nm among all CMD‐grafted Sepharose gels. A shrinkage of pore size was also typical of polymer‐grafted ion exchange adsorbents via grafting‐to and grafting‐from techniques [32, 33, 35, 39, 40]. Moreover, the result in Table 1 further indicated that chain length of CM dextran had marked influence on pore size of CMD‐grafted Sepharose gel. At the similar IC values, CMD10‐grafted Sepharose gel had a larger pore size than CMD40‐grafted Sepharose gel. For example, CMD40‐6FF‐IC260 gel had a smaller pore size than CMD10‐6FF‐IC250 even though both the gels had similar IC in Table 1. It was reported previously in dextran‐grafted gels by Stone *et al*. [44]. The difference in pore size meant distinct architecture of CMD10‐ and CMD40‐grafted layer on pore surface of both the gels and reflected different spatial arrangement of ion groups for protein binding. Stone *et al*. found that long dextran‐grafted ion exchange gels had higher adsorption capacity for IgG than short dextran‐grafted ion exchange gel even though both them had same ligand densities [44]. As shown in Figure S2, the higher amount of grafted CMD, the smaller the *K*_D_ value of the same probe molecule. Among these gels, CMD‐grafted layer has a greater influence on *K*_D_ values for those probe molecules with a radius of 2.99–9.40 nm. Stokes radius of IgG molecule (*r*_s,IgG_ = 4.22 nm) fell in this range [45].

**TABLE 1:** Physical properties of Sepharose 4FF, Sepharose 6FF and CMD‐grafted Sepharose gels

Resin	IC (mmol/L)	*d*_p_ (μm)	*ρ*_p_ (g/mL)	*r*_pore_ (nm)
Sepharose 4FF	0	89 ± 2	1.018 ± 0.004	21.26 ± 0.67
Sepharose 6FF	0	91 ± 3	1.020 ± 0.002	17.57 ± 0.49
CMD10‐6FF‐IC190	195 ± 4	94 ± 4	1.028 ± 0.005	14.76 ± 0.46
CMD10‐6FF‐IC250	252 ± 8	96 ± 3	1.034 ± 0.006	13.44 ± 0.75
CMD40‐6FF‐IC200	208 ± 9	95 ± 2	1.032 ± 0.004	14.46 ± 0.53
CMD40‐6FF‐IC260	265 ± 11	97 ± 3	1.035 ± 0.002	12.24 ± 0.91
CMD10‐4FF‐IC200	197 ± 13	90 ± 2	1.022 ± 0.004	17.57 ± 0.49
CMD10‐4FF‐IC250	254 ± 7	93 ± 3	1.026 ± 0.006	16.46 ± 0.35
CMD10‐4FF‐IC300	304 ± 11	95 ± 5	1.031 ± 0.008	15.78 ± 0.20
CMD10‐4FF‐IC400	399 ± 4	98 ± 4	1.042 ± 0.005	14.03 ± 0.48
CMD40‐4FF‐IC200	191 ± 15	91 ± 3	1.024 ± 0.003	16.74 ± 0.50
CMD40‐4FF‐IC250	246 ± 4	94 ± 4	1.032 ± 0.007	16.01 ± 0.31
CMD40‐4FF‐IC300	307 ± 12	97 ± 4	1.040 ± 0.004	15.76 ± 0.32
Sepharose 6B	–	–	–	19.4^a^
Sepharose 6B	–	–	–	18.2^c^
Sepharose 6FF	–	90 ± 2^b^	1.023 ± 0.002^b^	18.6^b^

^a^ The buffer was 10 mmol/L Na_2_HPO_4_ buffer (pH 6.5) and pH was adjusted using phosphoric acid [30].

^b^ The buffer was 1.0 mol/L NaCl in 10 mmmol/L Na2HPO4 buffer (pH 6.5) and pH was adjusted using phosphoric acid [30].

^c^ The buffer was 20 mmol/L Tris–HCl buffer (pH 8) [35].

**FIGURE 2:**
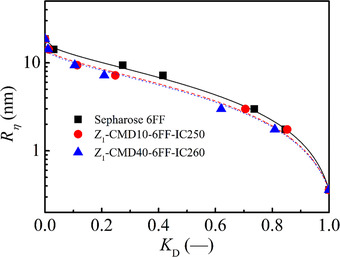
Dextran calibration curves for Sepharose 6FF and CMD‐grafted protein A gels using Sepharose 6FF matrix

After protein A ligands were coupled onto the CMD‐grafted Sepharose gels, pore radii of CMD‐grafted protein A gel were measured by iSEC. The representative dextran calibration curves are shown in Figure 2. It is obvious that those probes with a smaller viscosity radius corresponded to a larger *K*_D_ value whilst Z_1_‐CMD10‐6FF‐IC250 had a little larger *K_D_* value than Z_1_‐CMD40‐6FF‐IC260 gels. Based on a single cylindrical pore model, pore radii of both CMD‐grafted protein A gels were determined to be 14.73 ± 0.46 and 14.23 ± 0.55 nm, respectively. Both the pore radii were slightly larger than those of corresponding CMD‐grafted Sepharose gels in Table 1. Therefore, the coupling of protein A ligand did not lead to the collapse of CMD‐grafted layer in protein A gels and CMD‐grafted gel provided a stable framework for three‐dimensional arrangement of protein A ligands. Table S2 further revealed that pore radii of Z1‐CMD10‐6FF‐IC250 gel ranged from 13.60–14.88 nm in a range of salt concentrations from 0 to 150 mmol/L, suggesting that the spatial ligand arrangement was stable in experimental range. It is the important perquisite for effective utilization of protein A ligand in antibody binding.

### 3.2 IgG adsorption on CMD‐grafted protein A gels using Z_1_ ligand

**FIGURE 3:**
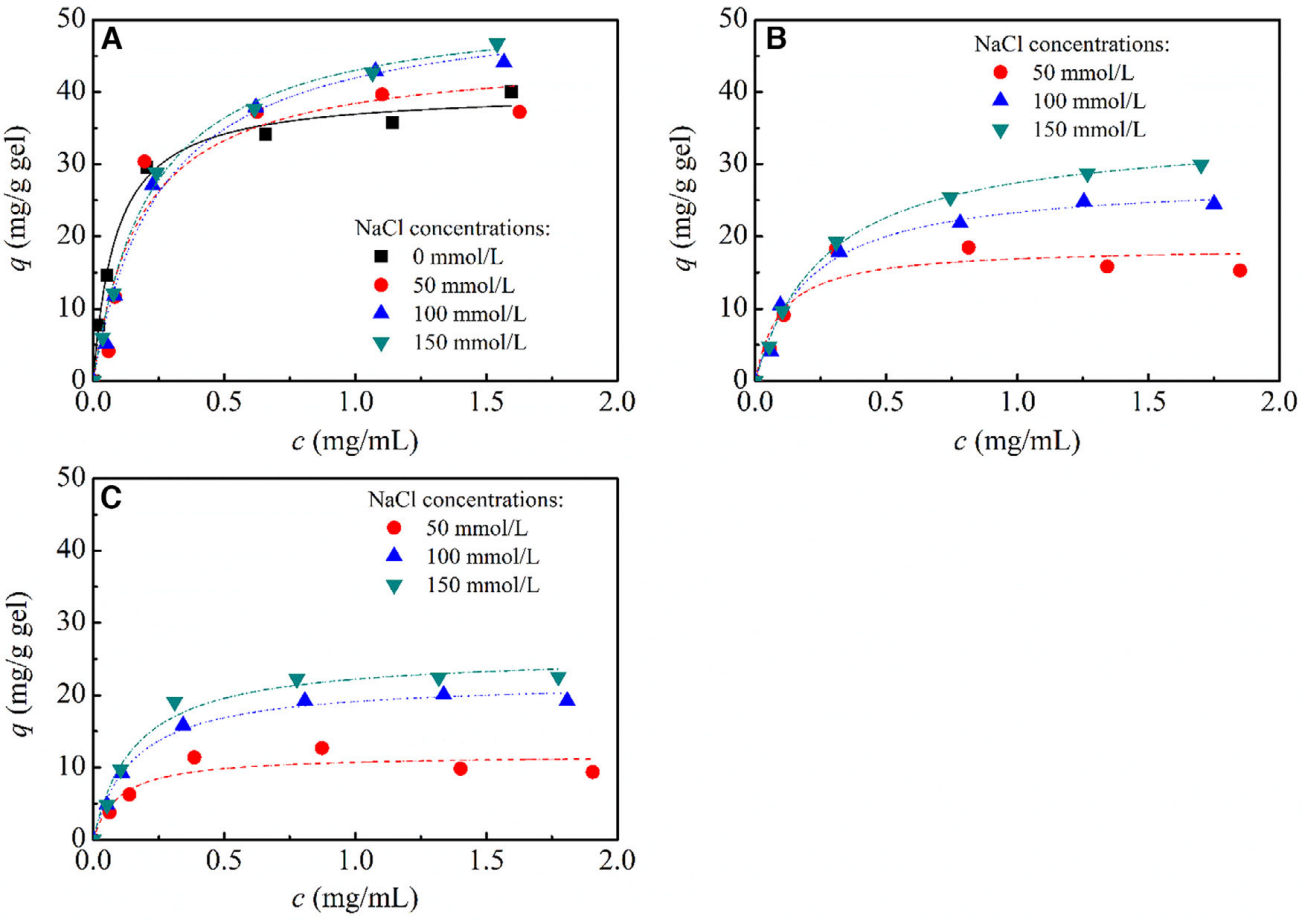
IgG adsorption on CMD‐grafted protein A gels and non‐grafted protein A gel at different salt concentrations. (A) Z_1_‐CMSep gel, (B) Z_1_‐CMD10‐6FF‐IC250 gel, and (C) Z_1_‐CMD40‐6FF‐IC260 gel

Figure 3 shows IgG adsorption on Z_1_‐CMD10‐6FF‐IC250 and Z_1_‐CMD40‐6FF‐IC260 as well as non‐grafted Z_1_‐CM Sepharose FF (Z_1_‐CMSep) at different salt concentrations and neutral pH (pH 7.4). With the help of zeta potential measurement at various buffer pH, zero point of zeta potential for IgG was determined to be pH 5.8, far from experimental pHs in this work [18]. Therefore, IgG had the charge with the same sign as carboxyl group on the surface of the gels at pH 7.4 and above, and contribution of electrostatic attraction to IgG binding could be ignored. In Figure 3, all the protein A gels exhibited higher adsorption amount of IgG with an increase of salt concentration while non‐grafted Z_1_‐CMSep had higher adsorption amount than CMD‐grafted protein A gels. With an increase in chain length, moreover, adsorption amount of IgG on CMD‐grafted protein A gels reduced. IgG adsorption on the three protein A gels was typical of Langmuir isotherm. Langmuir parameters are listed in Table 2. Among three protein A gels, non‐grafted Z_1_‐CMSep gel had maximal *q*_m_ of IgG at the same salt concentration and *q*_m_ values ranged from 40.2 to 55.9 mg/g gel. As stated previously by Wang *et al*. [20], at neutral pH, protein A ligand loss partial secondary structure on negative‐charged CMD‐coated surface. On CMD‐grafted protein A gels; therefore, the spatial carboxyl arrangement worsen the stability of secondary structure of protein A ligand and it led to a lower adsorption capacity. Such protein– surface interaction was extensively reported [46, 47, 48]. The influence of matrix surface to protein A ligand was further validated by an increased adsorption capacity with an increase of salt concentration in Table 2. An increased salt concentration reduced electrostatic attraction between protein A ligand and matrix surface and reshaped molecular structure of the ligand. At an increased salt concentration, therefore, molecular structure of protein A ligand was closer to its native structure, and benefit for IgG binding.

**TABLE 2:** Langmuir parameters of IgG adsorption on CMD‐grafted protein A gels and Z_1_‐CMSep gels

	Z_1_‐CMSep		Z_1_‐CMD10‐6FF‐IC250		Z_1_‐CMD40‐6FF‐IC260	
NaCl concentration (mmol/L)	*q*_*m*_ (mg/g gel)	*K*_*d*_ (mg/mL)	*q*_*m*_ (mg/g gel)	*K*_*d*_ (mg/mL)	*q*_*m*_ (mg/g gel)	*K*_*d*_ (mg/mL)
0	40.2 ± 1.1	0.088 ± 0.012	—	—	—	—
50	45.4 ± 1.0	0.182 ± 0.014	18.5 ± 1.1	0.092 ± 0.041	11.8 ± 1.3	0.097 ± 0.050
100	52.6 ± 2.5	0.256 ± 0.061	28.0 ± 1.4	0.199 ± 0.037	22.0 ± 0.7	0.151 ± 0.021
150	55.9 ± 0.3	0.270 ± 0.010	34.8 ± 0.6	0.270 ± 0.016	25.7 ± 1.2	0.157 ± 0.031

**FIGURE 4:**
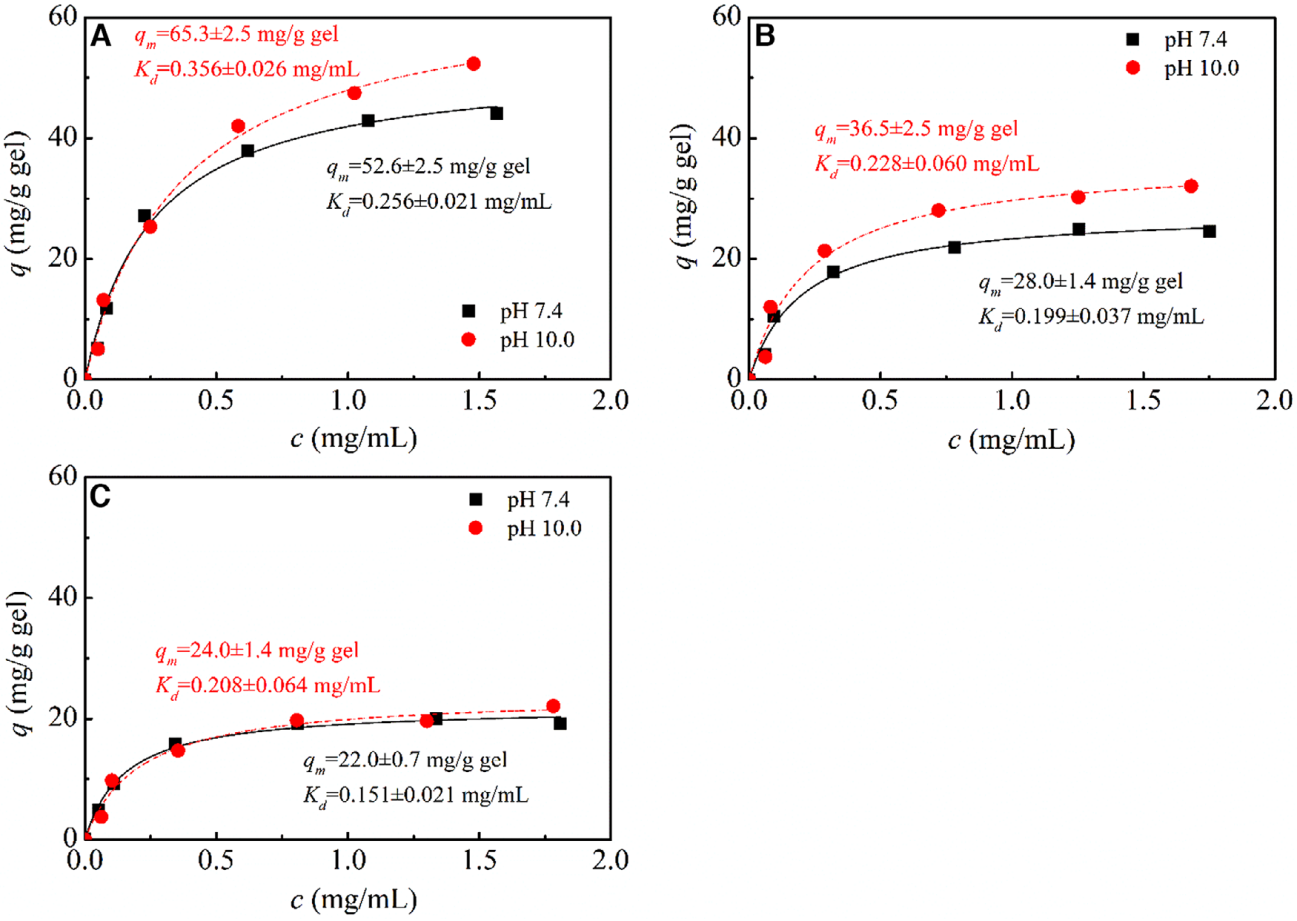
IgG adsorption on CMD‐grafted protein A gels and non‐grafted protein A gel at different buffer pHs. The experiment was conducted in 20 mmol/L phosphate buffer containing 100 mmol/L NaCl at pH 7.4 and 10.0. Protein A gels used in this work were (A) Z_1_‐CMSep gel, (B) Z_1_‐CMD10‐6FF‐IC250 gel, and (C) Z_1_‐CMD40‐6FF‐IC260 gel

Figure 4 shows IgG adsorption on CMD‐grafted protein A gels at a salt concentration of 100 mmol/L NaCl and different pHs. With an increase of buffer pH, adsorption amount of IgG on all protein A gels increased markedly. At buffer pH 10.0, *q_m_* for IgG increased by 24% on non‐grafted Z_1_‐CMSep gel while *q*_*m*_ for IgG increased more by 30% on CMD‐grafted Z_1_‐CMD10‐6FF‐IC250 gel. However, only a little increase in adsorption capacity was found in Z_1_‐CMD40‐6FF‐IC260 gel. As mentioned by Wang *et al*. [20], protein A ligand maintained intact secondary structure on negative‐charged surface at pH 10.0. Stable structure of protein A ligand improved IgG binding and a higher adsorption capacity was obtained at pH 10.0. It can be confirmed that, on CMD‐grafted protein A gels, protein A ligand reshaped its molecular structure with an increase in buffer pH and maintained intact molecular structure at pH 10.0, thereby improving IgG adsorption.

### 3.3 Influence of dextran layer in IgG adsorption on CMD‐grafted protein A gels

**FIGURE 5:**
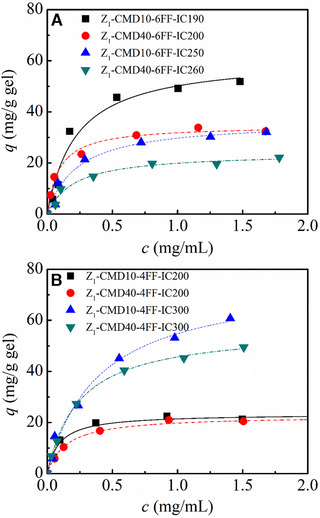
IgG adsorption on CMD‐grafted protein A gels with different molecular weight and grafted amount of CMD. (A) Sepharose 6FF based CMD‐grafted protein A gels, (B) Sepharose 4FF based CMD‐grafted protein A gels

Figure 5 shows the influence of dextran layer in IgG adsorption on CMD‐grafted protein A gels using two matrices, Sepharose 6FF and Sepharose 4FF. CMD grafting provides a three‐dimensional framework for the immobilization of protein A ligands. With a decrease in IC, pore radii of CMD‐grafted gels increased in Table 1. It corresponded to a decrease in the depth of dextran layer. For those Sepharose 6FF based protein A gels in Figure 5A, a decrease in IC and chain length of CMD improved IgG adsorption on CMD‐grafted protein A gels. However, IgG adsorption exhibited a distinct behavior on those Sepharose 4FF based protein A gels in Figure 5B. A decrease in IC led to a reduced adsorption amount of IgG. In contrast, a decrease in chain length of CMD increased IgG adsorption. It was more significant at a higher IC value corresponding to higher grafted amount of CMD. It may be attributed to a striking difference between two Sepharose gels that Sepharose 4FF gel had a larger pore size than Sepharose 6FF gel as listed in Table 1. Langmuir parameters of IgG adsorption are listed in Table 3. The result indicated that an increase in chain length of CMD from 10 to 40 kDa brought about an adverse effect on *q*_m_. Moreover, Gu *et al*. found that a further decrease of chain length from 20 k (T20) to 5 k (T5) was likely unfavorable for the synthesis of high‐capacity mixed‐mode gels and resultant dextran T5‐grafted mixed mode gel had similar adsorbed amount for proteins to the non‐grafted gels [34]. Among CMD‐grafted protein A gels in this work, the maximal value of *q*_m_ was obtained to be 76.3 mg/g gel on Z_1_‐CMD10‐4FF‐IC300 gel as listed in Table 3. Therefore, it suggests that a short dextran, CMD10, is a more suitable “spatial scaffold” for protein A ligand and the resultant protein A gels had higher adsorption capacity for IgG.

**TABLE 3:** Ligand densities and Langmuir parameters of CMD‐grafted protein A gels based on Sepharose 6FF and Sepharose 4FF

Gel	Ligand density (mg/g gel)	*q*_*m*_ (mg/g gel)	*K*_*d*_ (mg/mL)
Z_1_‐CMD10‐6FF‐IC190	8.7	61.3 ± 5.1	0.356 ± 0.026
Z_1_‐CMD40‐6FF‐IC200	8.6	34.7 ± 1.1	0.090 ± 0.015
Z_1_‐CMD10‐6FF‐IC250	9.6	36.5 ± 2.5	0.228 ± 0.060
Z_1_‐CMD40‐6FF‐IC260	9.3	24.0 ± 1.4	0.208 ± 0.064
Z_1_‐CMD10‐4FF‐IC200	8.8	23.4 ± 1.0	0.091 ± 0.019
Z_1_‐CMD40‐4FF‐IC200	9.0	22.7 ± 0.7	0.146 ± 0.018
Z_1_‐CMD10‐4FF‐IC300	9.8	76.3 ± 5.2	0.393 ± 0.075
Z_1_‐CMD40‐4FF‐IC300	9.1	56.8 ± 0.8	0.245 ± 0.015

**FIGURE 6:**
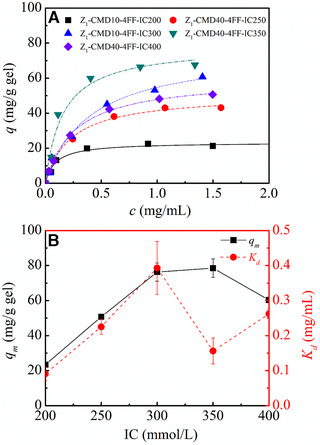
IgG adsorption on CMD‐grafted protein A gels with different grafted amount of CMD10 based on Sepharose 4FF

**FIGURE 7:**
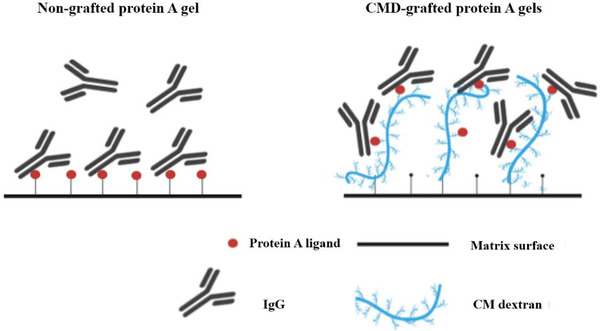
Schematic of IgG adsorption in non‐grafted and CMD‐grafted protein A gels

Figure 6 shows the influence of grafted amount of CMD10 to IgG adsorption on Sepharose 4FF based protein A gels. In Figure 6A, adsorption amount of IgG increased with an increase of IC to 350 mmol/L. However, a further increase in IC to 400 mmol/L led to a great decrease in adsorption amount. Langmuir parameters of CMD10‐grafted protein A gels are shown in Figure 6B. At the similar ligand density, *q*_*m*_ values increased by over 220% as IC increased from 200 mmol/L to 300–350 mmol/L. In this case, IgG binding was dominant by affinity interaction with protein A ligand because electrostatic attraction between IgG and negative‐charged matrix surface could completely be ignored at buffer pH much higher than p*I* of IgG [18]. A further increase in IC led to a little decrease of *q*_*m*_ to 60.3 mg/g gel on Z_1_‐CMD10‐4FF‐IC400 gel. The influence of IC to *q*_*m*_ was also reported in polymer‐grafted ion exchange gel by Yu *et al*. [39]. In poly(ethylenimine)‐grafted Sepharose FF, *q*_*m*_ likely showed a trend of initial increasing and then decreasing with an increase in IC and the maximal value of *q*_m_ was obtained at IC = 740 mmol/L. It should be worthy to note that carboxyl group on CMD‐grafted Sepharose gel had different arrangement from non‐grafted CM Sepharose gel. At the similar IC, cluster distribution of carboxyl group along dextran chain led to non‐uniform arrangement of protein A ligand. It was different from that on non‐grafted protein A CMSep gel as presented in Figure 7. At low grafted amounts of CMD, therefore, the ligand utilization on CMD‐grafted protein A gel was lower. With an increase of grafted amount of CMD, the ligand utilization on CMD‐grafted protein A gel increased in Figure 7. As shown in Figure 6B, an optimal amount of grafted CMD ranging from 300 to 350 mmol/L was applied to synthesize CMD‐grafted protein A gel.

### 3.4 Influence of protein A ligand in IgG adsorption on CMD‐grafted protein A gels

**FIGURE 8:**
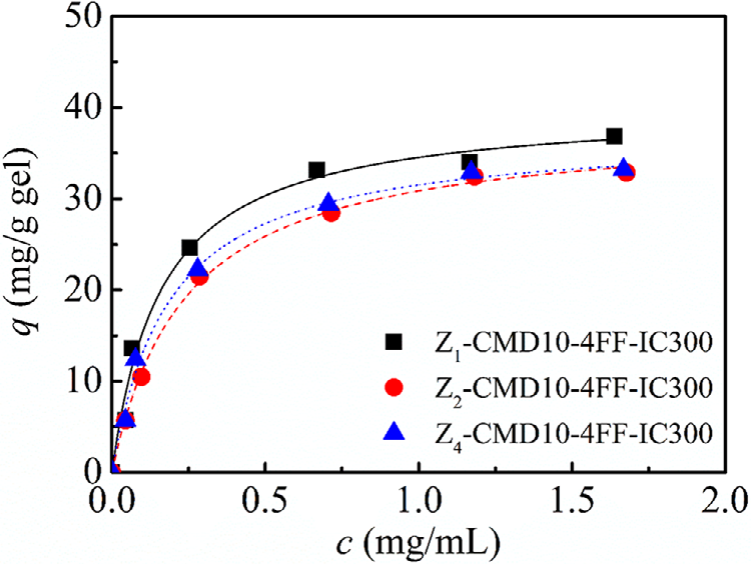
IgG adsorption on CMD‐grafted protein A gels coupling respectively with Z_1_, Z_2_, and Z_4_ ligands

Figure 8 shows IgG adsorption on CMD‐grafted protein A gels coupling, respectively, with Z_1_, Z_2_ and Z_4_ ligands. At the similar mass density of protein A ligand (∼3.8 mg/g gel), three CMD‐grafted protein A gels coupling, respectively with Z_1_, Z_2_ and Z_4_ ligands had similar adsorption amount, indicating the similar utilization of binding sites on protein A ligand to IgG binding. Previously, von Raman *et al*. reported that adsorption capacity for IgG increased with an increase in polymerization degree of the ligand at the similar molar density [17]. The phenomenon was also reported by Müller and Vajda [14]. In this work, at the ligand density of ∼3.8 mg/g gel, adsorption capacities were determined to be 40.1 mg/g gel on Z_1_‐CMD10‐44F‐IC300 gel, 38.2 mg/g gel on Z_2_‐CMD10‐44F‐IC300 gel and 37.3 mg/g gel on Z_4_‐CMD10‐44F‐IC300 gel, respectively. Among three CMD‐grafted protein A gels; however, Z_1_‐CMD10‐44F‐IC300 gel had the highest affinity to IgG and maximal value of *K_d_* was determined to be 0.161 mg/mL. The result revealed that protein A ligands coupled on dextran layer shielded steric hindrance effect of matrix surface to the ligand and improved the utilization of protein A ligand.

**FIGURE 9:**
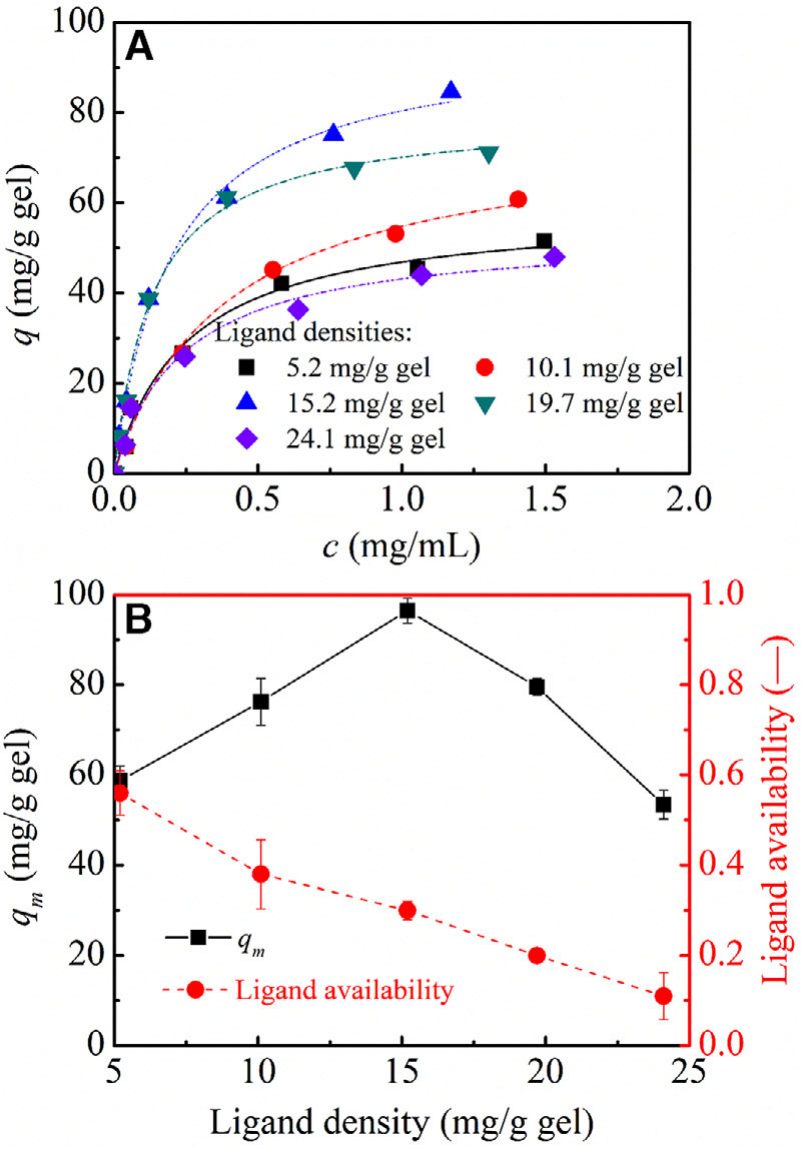
IgG adsorption on CMD‐grafted protein A gels at different ligand densities. (A) adsorption isotherms, (B) adsorption capacity for IgG and ligand availability CMD‐grafted protein A gels

The influence of ligand density on IgG adsorption is shown in Figure 9. It was seen that ligand density had a great influence on IgG binding and maximal adsorption amount was observed at a ligand density of 15 mg/g gel. Figure 9B further revealed that *q*_*m*_ value reached to 96.4 mg/g gel at a ligand density of 15 mg/g gel. With a further increase in ligand density, adsorption capacity decreased markedly, and minimal adsorption capacity was determined to be 53.4 mg/g gel at the highest ligand density of 24.1 mg/g gel. At the same time, ligand availability decreased greatly with an increase in ligand density as shown in Figure 9B. A decreased ligand availability was also found previously by Yang *et al*. on non‐grafted protein A gels [18]. At low ligand density, *q*_m_ and ligand availability of Z_1_‐CMD10‐4FF‐IC300 were higher than that of Z_1_‐CM Sepharose, which can be considered that the three‐dimensional binding space provided by the dextran graft layer did have a positive effect on reducing steric hindrance.

### 3.5 Dynamic binding capacity of IgG

**FIGURE 10:**
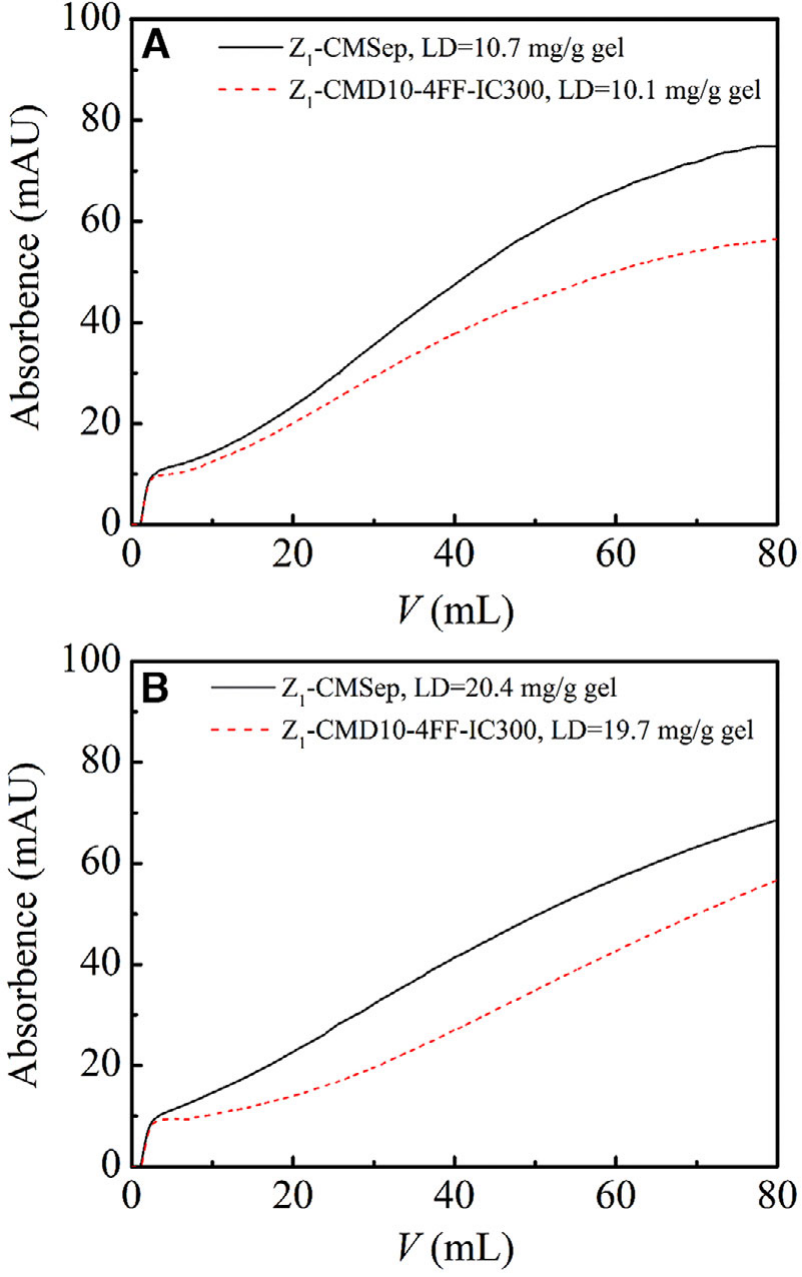
Breakthrough curves of non‐grafted Z_1_‐CMSep gel and Z_1_‐CMD10‐4FF‐IC300 gel at different ligand densities. The ligand densities (LDs) were (A) 10 mg/g gel and (B) 20 mg/g gel

Figure 10 shows the breakthrough of IgG on non‐grafted Z_1_‐CMSep gel and Z_1_‐CMD10‐4FF‐IC300 gel at a flow rate of 0.5 mL/min. Due to the complexity of composition in commercially available IgG, unbound trace in sample solution flew through protein A column quickly and a small platform of 10 mAU was recorded. Similar phenomenon was observed in our previous work [18]. In the breakthrough experiment, dynamic binding capacity was calculated at 10% breakthrough. Figure 10 showed that, at the outlet, IgG concentration increased more quickly in Z_1_‐CMSep gels than CMD‐grafted protein A gels. At a ligand density of 10 mg/g gel, DBC of Z_1_‐CMSep gel was determined to be 20.2 mg/mL and that of Z_1_‐CMD10‐4FF‐IC300 gel was 27.2 mg/mL, indicating that DBC of CMD‐grafted increased by 34% in CMD‐grafted protein A gel. At the same time, the values of *q*_*m*_ were 65.3 mg/g gel for Z_1_‐CMSep gel and 76.2 mg/g gel for Z_1_‐CMD10‐4FF‐IC300 gel at the ligand density of 10 mg/g gel. The ratios of DBC to *q*_m_ were 31% for non‐grafted protein A gel and 36% for CMD‐grafted protein A gel. At the ligand density of 20 mg/g gel, DBC of Z_1_‐CMD10‐4FF‐IC300 gel was 32.8 mg/mL and that of Z_1_‐CMSep gel was 27.0 mg/mL. At the same time, the values of *q*_*m*_ were 80.7 mg/g gel for Z_1_‐CMSep gel and 79.0 mg/g gel for Z_1_‐CMD10‐4FF‐IC300 gel. The ratios of DBC to *q*_*m*_ were 33% for non‐grafted protein A gel and 41% for CMD‐grafted protein A gel. The result of DBC exhibited a higher capacity utilization in CMD‐grafted protein A gel than non‐grafted protein A gel. It was worthy to note that it was achieved at higher flow rate, at which intraparticle mass transfer became worsen and DBC of protein A gel decreased.

## 4 CONCLUDING REMARKS

In this work, CMD was introduced to provide a stable polymer layer for the coupling of protein A ligand to improve IgG adsorption on protein A gels. The result showed that coupled protein A ligand in CMD‐grafted layer formed a stable spatial arrangement of the ligand in pores of two Sepharose gels, which was characterized with a striking feature of pore shrinkage of Sepharose gels. IgG adsorption on CMD‐grafted protein A gels was typical of Langmuir isotherms, and adsorption capacity for IgG increased greatly with an increase in salt concentration and buffer pH in the experimental range. At the same IC values, adsorption capacity for IgG reduced with an increase in molecular weight of CMD, suggesting that CMD10 was more suitable as a scaffold for the synthesis of high‐capacity protein A gels. With an increase in IC values, moreover, Sepharose 4FF‐based CMD‐grafted protein A gels could obtain higher adsorption capacities for IgG and the maximal value of capacity was found at IC values of 300–350 mmol/L. In contrast, adsorption capacity for IgG decreased with an increase in IC on Sepharose 6FF‐based CMD‐grafted protein A gels. By optimizing the synthesis of CMD‐grafted protein A gels, the maximum value of adsorption capacity for IgG was obtained to be 96.4 mg/g gel at a ligand density of 15.2 mg/g gel for Z_1_ ligand. A further increase in ligand density led to a significant decrease in adsorption capacity in ligand availability. The result of DBC exhibited a higher capacity utilization in CMD‐grafted protein A gel than non‐grafted protein A gel. The research in this work made a valuable attempt to establish a stable dextran layer for the development of polymer‐grafted protein A gels with high adsorption capacity and demonstrated the importance of stable polymer layer to improve binding capacity for IgG in polymer‐grafted protein A gels.

## CONFLICT OF INTEREST

The authors have declared no conflict of interest.

## Supporting information

Supporting Information

## Data Availability

The data that support the findings of this study are available from the corresponding author upon reasonable request.
