# Supplementary material for: Increasing immunoglobulin G adsorption in dextran‐grafted protein A gels
Source: Eng Life Sci. 2021 Mar 20;21(6):392–404. doi: 10.1002/elsc.202000097 (PMC8182282; doi:10.1002/elsc.202000097)
Supplement: Supplementary file 1 — Supporting Information [file ELSC-21-392-s001.pdf]

Supporting information

Increasing Immunoglobulin G adsorption in dextran-grafted protein A gels

Limin Huan[1], Qing-Hong Shi[1,2]

[1]Department of Biochemical Engineering, School of Chemical Engineering and Technology, Tianjin University, Tianjin 300350, China

[2]Key Laboratory of Systems Bioengineering and Frontiers Science Center for Synthetic Biology (Ministry of Education), Tianjin University, Tianjin 300350, China

Table 1 Average molecular weights (M_w), and viscosity radii (R_η) of glucose and dextran standards used for iSEC experiments

Dextran	M_w (Da)	R_η (nm)
Glucose	180.16	0.36
Dextran 3.6k	3620	1.60
Dextran 12.6k	12,600	2.99
Dextran 70.8k	70,800	7.05
Dextran 126k	126,000	9.40
Dextran 289k	289,000	14.21
Dextran 496k	496,000	18.59
Blue dextran	2,000,000	37.20

Table S2 Average pore radii of Z_1-CM10-6FF-IC250 at different salt concentrations

gel	NaCl （mmol/L）	r_{pore} (nm)
Z_1-CMD10-6FF-IC250	0	13.60±0.75
	50	14.32±0.61
	100	14.73±0.46
	150	14.88±0.60

Z_1: HHHHHH VDNKF NKEQQ NAFYE ILHLP NLNEE QRNAF IQSLK DDPSQ SANLL AEAKK LNDAQ APKCK

Z_2: HHHHHH VDNKF NKEQQ NAFYE ILHLP NLNEE QRNAF IQSLK DDPSQ SANLL AEAKK LNDAQ APK GGGG VDNKF NKEQQ NAFYE ILHLP NLNEE QRNAF IQSLK DDPSQ SANLL AEAKK LNDAQ APKCK

Z_4: HHHHHH VDNKF NKEQQ NAFYE ILHLP NLNEE QRNAF IQSLK DDPSQ SANLL AEAKK LNDAQ APK GGGG VDNKF NKEQQ NAFYE ILHLP NLNEE QRNAF IQSLK DDPSQ SANLL AEAKK LNDAQ APK GGGG VDNKF NKEQQ NAFYE ILHLP NLNEE QRNAF IQSLK DDPSQ SANLL AEAKK LNDAQ APK GGGG VDNKF NKEQQ NAFYE ILHLP NLNEE QRNAF IQSLK DDPSQ SANLL AEAKK LNDAQ APKCK

Figure S1 Amino acid sequences of monomeric (Z_1), dimeric (Z_2) and tetrameric domains (Z_4)

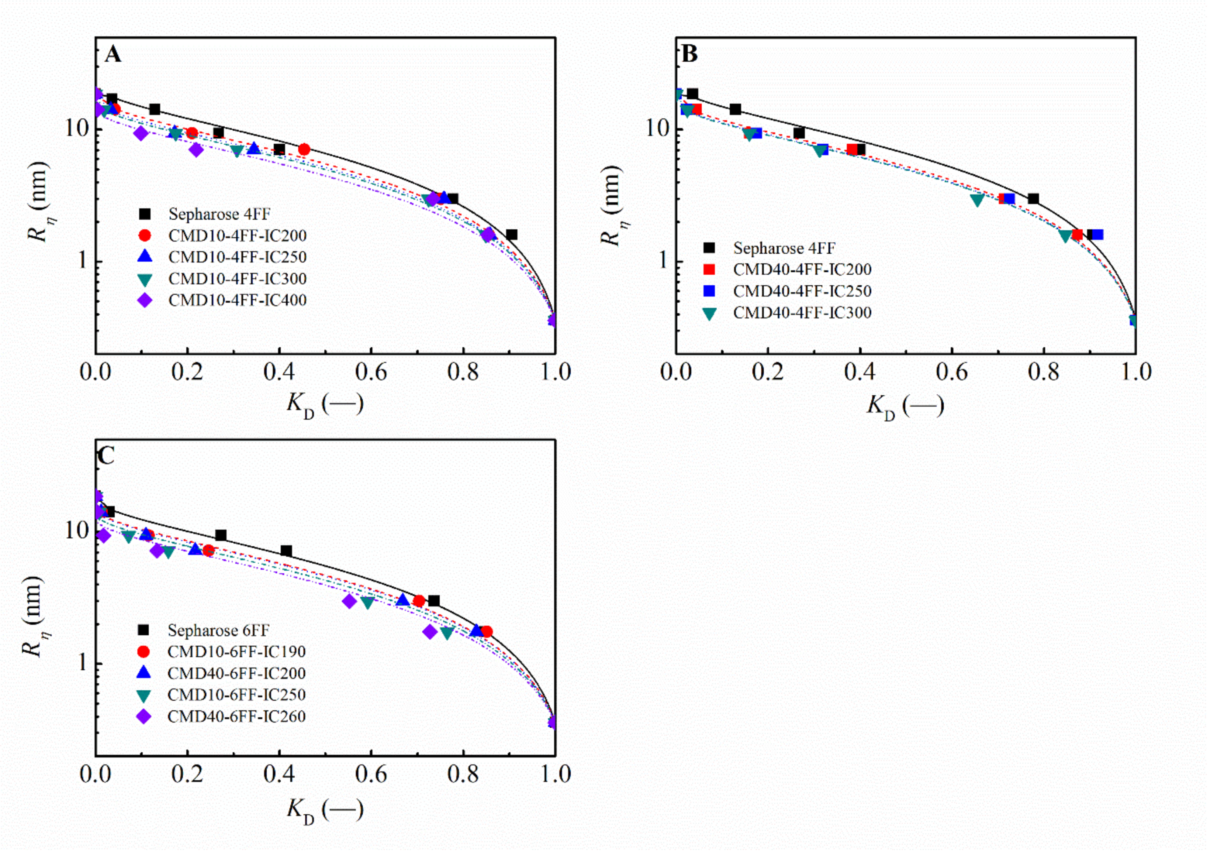

Figure S2 Dextran calibration curves for Sepharose 4FF, Sepharose 6FF and CMD-grafted Sepharose gels (A) Sepharose 4FF and CMD10-4FF gels, (B) Sepharose 4FF and CMD40-4FF gels; (C) Sepharose 6FF, CMD10-6FF and CMD40-6FF gels
